# Pressure Dynamic Characteristics of Pressure Controlled Ventilation System of a Lung Simulator

**DOI:** 10.1155/2014/761712

**Published:** 2014-08-13

**Authors:** Yan Shi, Shuai Ren, Maolin Cai, Weiqing Xu, Qiyou Deng

**Affiliations:** ^1^School of Automation Science and Electrical Engineering, Beihang University, Beijing 100191, China; ^2^The State Key Laboratory of Fluid Power Transmission and Control, Zhejiang University, Hangzhou 310058, China; ^3^Department of Mechanical Engineering, University of Bath, Bath BA2 7AY, UK

## Abstract

Mechanical ventilation is an important life support treatment of critically ill patients, and air pressure dynamics of human lung affect ventilation treatment effects. In this paper, in order to obtain the influences of seven key parameters of mechanical ventilation system on the pressure dynamics of human lung, firstly, mechanical ventilation system was considered as a pure pneumatic system, and then its mathematical model was set up. Furthermore, to verify the mathematical model, a prototype mechanical ventilation system of a lung simulator was proposed for experimental study. Last, simulation and experimental studies on the air flow dynamic of the mechanical ventilation system were done, and then the pressure dynamic characteristics of the mechanical system were obtained. The study can be referred to in the pulmonary diagnostics, treatment, and design of various medical devices or diagnostic systems.

## 1. Introduction

As an important life-saving treatment, mechanical ventilation is adopted to ventilate patients who cannot breathe adequately on their own [1]. The most commonly used ventilation technique today is positive pressure mechanical ventilation, which is applied by using many different modalities [1–3]. Pressure controlled ventilation (PCV) is an alternative mode of ventilation, which is widely used in severe respiratory failure [4–14]. In recent studies, it was approved that PCV can improve arterial oxygenation and decrease peak airway pressure due to its decelerating inspiratory flow [4, 9, 14–16]. However, PCV has its shortages, such as inadequate ventilation and hyperventilation [17–20].

Nowadays, dynamic characteristics of respiratory system and models of different medical conditions are referred to in pulmonary diagnostics and treatments [21–29]. However, mechanical respiratory properties cannot be measured directly; therefore, they are generally represented by means of lumped parameters, such as the overall breathing resistance, *R*, and compliance, *C* [30].

Because of the physical analogies between pneumatic and electrical systems, the structure of the human respiratory tract is usually presented as analogous to an electrical system [31–36]. This electrical system consists of the RIC elements, which represent the pneumatic mechanical resistances, inertances, and compliances of specific anatomical parts [32]. These models have some advantages, such as simplicity and no requirement for significant computing power. However, they have many serious shortages too; for example, it cannot change the nature and the intensity of lesions modeled, which significantly reduces their versatility and applicability, seriously influences optimization algorithms on the effectiveness and accuracy of parameter identification, and cannot efficiently identify reliability obtained for certain model parameters [21, 32–36].

To illustrate the pressure dynamic characteristics of PCV mechanical ventilation system, in this paper, first of all, mechanical ventilation system is considered as a pure pneumatic system, which has a better versatility and applicability, and may improve the effectiveness and accuracy of parameter identification.

Furthermore, based on the equivalent pneumatic system, a mathematical model of PCV mechanical ventilation system is set up, and influence of parameters on respiratory resistance (*R*) is analyzed.

Moreover, to verify the mathematical model and void injury to real lung, a prototype PCV mechanical ventilation system of a lung simulator is proposed. On the basis of experimental and simulation study on the prototype system, its dynamic characteristic can be obtained and analyzed.

Last, influences of key parameters on the pressure dynamic characteristics are studied.

## 2. Introduction and Modeling of Mechanical Ventilation System

### 2.1. Introduction of Mechanical Ventilation System

**Figure 1:**
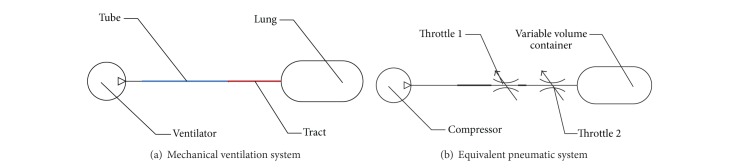
Structures of mechanical ventilation system and equivalent pneumatic system.

A typical simplified mechanical ventilation system, as shown in Figure 1(a), consists of a ventilator, a flexible tube, a respiratory tract, and a human lung. Positive pressure ventilation is made by the ventilator, to force airflow into the human lung, and then an inspiration process is accomplished. After the inspiration, because of the elasticity of the human lung, air is expelled from the human lung to the atmosphere, through an exhalation valve embedded in the ventilator, and then an expiration process is completed.

According to the function of the ventilator, it can be regarded as an air compressor.

Efficiency of ventilation depends on the matching of ventilator settings to the actual mechanical properties of the respiratory system, which mainly consist of respiratory resistance (*R*_*r*_) and compliance (*C*) [31].

In this simplified system, the respiratory resistance (*R*_*r*_), which varies with time, is basically represented as friction loss in the tube and the respiratory tract. Therefore, the tube and respiratory tract can be considered as two equivalent throttles.

The respiratory compliance (*C*), which varies with time too, is so complicated that human lung can be considered as a variable volume container.

Because the pressure of ventilation system is about 2 cm H_2_O~40 cm H_2_O, respiratory compliances (*C*) of the tube and respiratory tract can be neglected [37–39]. Therefore, a mechanical ventilation system can be equivalent to a pure pneumatic system, as shown in Figure 1(b). The compressor, the container, and throttles 1, 2 represent ventilator, human lung, tube, and respiratory tract, respectively.

### 2.2. Mathematical Modeling of Mechanical Ventilation System

According to the working principle of mechanical ventilation system, its working process can be considered as inflation and deflation of a variable volume container. To facilitate research, the following assumptions are made [40]:

1. air of the system follows all ideal gas laws;
2. the temperature, pressure, and density field of air in the same capacity are uniform. At any time, state parameter of air anywhere in the capacity is the same; the dynamic process is quasi-balanced process;
3. there is no air leakage during the working process;
4. in each moment of dynamic process, the flow state of air is the same as the state of steady flow under the same conditions;
5. the flow of air flowing into and out of the lung simulator is stable one-dimensional flow, equivalent to the flow of air through the nozzle contraction.

#### 2.2.1. Flow Equation

When air flows through throttle, its mass flow can be calculated by the equation when air flows through the LAVAL nozzle. When *p*_*d*_/*p*_*u*_ > *b*, air flow is subsonic and when *p*_*d*_/*p*_*u*_ ≤ *b*, air flow is sonic. As the pressure of the prototype ventilation system is almost 2 cm H_2_O~40 cm H_2_O, therefore, *p*_*d*_/*p*_*u*_ is always bigger than *b*, so mass flow equations of mechanical ventilation system can be obtained by

(1) $$\begin{array}{c} q=A_{e}P_{u}\frac{1}{\sqrt{T}}\sqrt{\frac{2\kappa }{\left(\kappa -1\right)}\cdot \frac{1}{R}\left[{\left(\frac{P_{d}}{P_{u}}\right)}^{2/\kappa }-{\left(\frac{P_{d}}{P_{u}}\right)}^{\left(\kappa +1\right)/\kappa }\right]}, \end{array}$$

where *n*_*i*_ is flow coefficient; it is 1 when air flows into chamber. Inversely, it is −1 when air is exhausted from chamber.

In this study, air temperature is constant and equal to atmosphere temperature, *R* is 287, and *b* is 0.528. In the standard reference atmosphere state, (1) can be approximately written as

(2) $$\begin{array}{c} q=0.0048\times n_{i}A_{e}p_{u}\sqrt{\frac{p_{d}}{p_{u}}\left(1-\frac{p_{d}}{p_{u}}\right)}. \end{array}$$

Volume flow of air can be calculated by the following equation:

(3) $$\begin{array}{c} Q=0.004\times n_{i}A_{e}p_{u}\sqrt{\frac{p_{d}}{p_{u}}\left(1-\frac{p_{d}}{p_{u}}\right)}. \end{array}$$

#### 2.2.2. Pressure Equation

The prototype ventilation system can be assumed as an isothermal system; the differential expression of Clapeyron equation (*PV* = *mRT*) can be given:

(4) $$\begin{array}{c} \frac{dp}{dt}=\frac{1}{V}RTq-mRT\frac{1}{V^{2}}\frac{dV}{dt},\\ \frac{dp}{dt}=\frac{RTqV}{V^{2}+CmRT}. \end{array}$$

#### 2.2.3. Volume Equation

According to the definition of respiratory compliance (*C*), the compliance (*C*) of the lung can be described as [11]

(5) $$\begin{array}{c} C=\frac{dV}{dp}. \end{array}$$

Then, the volume of the lung can be calculated by the following formula:

(6) $$\begin{array}{c} dV=Cdp. \end{array}$$

#### 2.2.4. Resistance Equation

Based on the assumptions above, the resistance *R*_*r*_ of the ventilation system can be given by [41, 42]

(7) $$\begin{array}{c} R_{r}=\frac{8\lambda \rho lQ}{\pi^{2}d^{5}}=\frac{8\lambda lq}{\pi^{2}d^{5}}, \end{array}$$

where *λ* is friction coefficient, which is determined by Reynolds number of air and relative roughness.

The difference between the output pressure of the ventilator and the pressure in the lung is defined as the pressure loss (*p*_loss_) of the ventilation system, and it can be gotten by

(8) $$\begin{array}{c} p_{\text{loss}}=R_{r}Q=\frac{8\lambda lqQ}{\pi^{2}d^{5}}. \end{array}$$

In the study, the pipe is plastic and its maximum diameter is 22 mm, and therefore according to [43], the relative roughness of the pipe and throttle is less than 3.125∗10^−4^. According to Moody diagram, *λ* can be calculated by

(9) $$\begin{array}{c} \lambda =\{\begin{array}{cc} 0.045-\frac{R_{e}-3000}{10000}\times 0.015 & R_{e}>3000\\ 0.032+\frac{R_{e}-2000}{1000}\times 0.013 & 3000\geq R_{e}\geq 2000\\ \frac{64}{R_{e}} & R_{e}<2000. \end{array} \end{array}$$

According to (2), *q* is determined by *A*_*e*_ (namely, *πd* ^2^/4), *p*_*u*_, and *p*_*d*_. Therefore, the resistance *R*_*r*_ is determined by *l*, *d*, *p*_*u*_, and *p*_*d*_. In clinical operation, lengths of tube and respiratory tract are almost constant. So, when *p*_*u*_ and *p*_*d*_ are fixed, *R*_*r*_ is only affected by *d*, which can be used to represent resistance.

## 3. Experimental and Simulation Study

### 3.1. Experimental Apparatus

In this study, to avoid injury to real lung, a lung simulator is adopted. The inlet diameter of the lung simulator is just 3.2 mm, and then it can be considered as a combined throttle of equivalent throttles 1 and 2, as shown in Figure 1.

**Figure 2:**
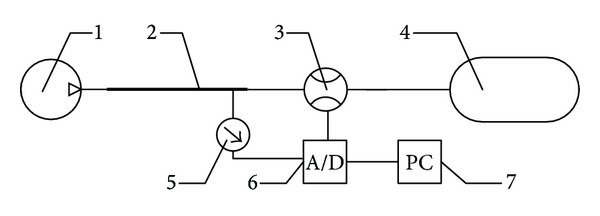
Configuration of experimental apparatus. (1) Ventilator, (2) tube, (3) flow sensor, (4) lung simulator, (5) pressure sensor, (6) data acquisition card, and (7) computer.

The experimental apparatus, shown in Figure 2, consists of a ventilator, a tube, a flow sensor, a pressure sensor, a lung simulator, a data acquisition card, and a computer. The adopted flow sensor and pressure sensor combination is an air power meter (APM-450) by Tokyo Meter, which can measure the pressure, flow, and temperature of compressed air, simultaneously. The uncertainty of the pressure, flow, and temperature is 0.1%, ±1% F.S., and 0.1°C, respectively [44, 45].

In this experiment, firstly, we open the ventilator and adjust the ventilator settings to the fixed value. When the ventilation system works steadily, we execute data acquisition and preservation.

### 3.2. Experimental Study

Because the lung simulator is a passive lung simulator, the adopted model of ventilation is pressure controlled model (PCV). The values of the main ventilator settings, including inspiratory positive airway pressure (IPAP), expiratory positive airway pressure (EPAP), breaths per minute (BPM), inspiratory time (*T*_*i*_), and rise time of pressure (*T*_*r*_), are shown in Table 1. The experiment can be performed according to the method described above.

The fluctuation amplitude of air flow and pressure is so large and the frequency is so high that wavelet filter technology was adopted in this study [46].

Through the experiment, it can be calculated that the compliance (*C*) of the lung simulator is about 10 mL/cm H_2_O.

### 3.3. Simulation of the Ventilation System

As the output dynamic of the ventilator is unassured and cannot be simulated exactly, in order to acquire precise simulation results, the output pressure of the ventilator is fitted, and the fitted output pressure is used as input pressure of tube, which connects to lung simulator.

In addition, the diameter (22 mm) of the tube is far greater than the inlet diameter (3.2 mm) of lung simulator; therefore, the respiratory resistance of the ventilation system mainly results from the resistance of the inlet of lung simulator, and the resistance due to the tube can be neglected.

The initial values of the parameter in simulation are the same as the values in experiment. The software, MATLAB/Simulink, is used for simulation.

### 3.4. Analysis and Discussions

**Figure 3:**
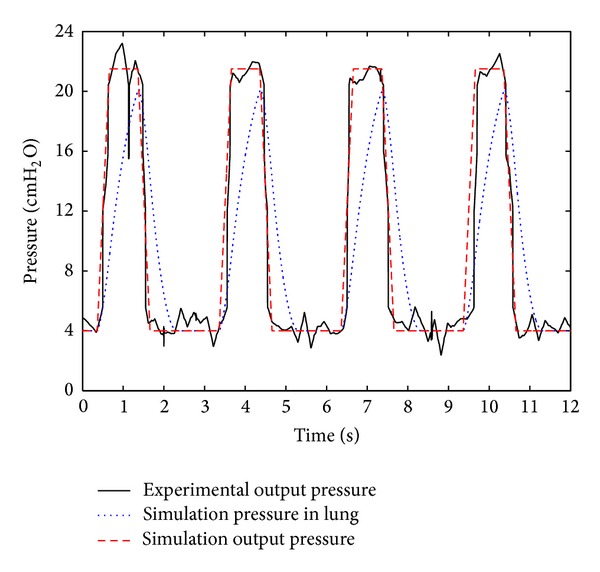
Curve and fitted curve of air pressure in tube.

**Figure 4:**
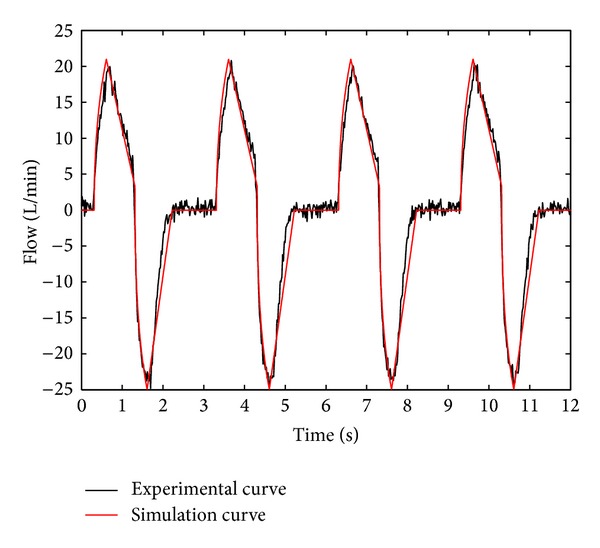
Curve of air flow in the system.

**Figure 5:**
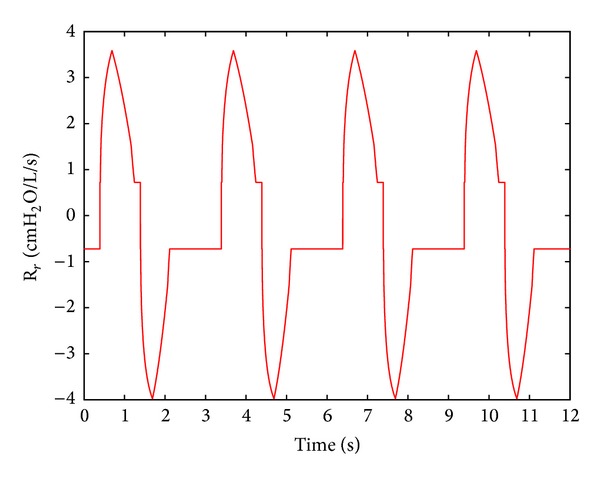
Respiratory resistance of the system.

The curve and fitted curve of output pressure of the ventilator as well as the curve of the air pressure in the lung simulator are shown in Figure 3. The air flow of the respiratory system and the respiratory resistance of the system, which are obtained by experimentation and simulation, are shown in Figures 4 and 5. The experimental output pressure curve is the output pressure of the ventilator in the experimental study; the simulation output pressure curve is the output pressure of the ventilator in the simulation study; the simulation pressure in lung curve is the pressure in the lung simulator in the simulation study.

From Figure 3, the following can be summarized.

1. As the average IPAP and EPAP, in the report by the ventilator, are 21.3 cm H_2_O and 3.9 cm H_2_O, respectively, hence, the measured data are consistent with the ventilator report, and the experiment results are authentic and reliable.
2. With a growth in the output pressure of the ventilator, the air pressure in the lung simulator rises. However, when the output pressure of the ventilator reaches the top flat, the air pressure in the lung simulator continues to rise, until it is equal to the output pressure of the ventilator. After that, the air pressure in the lung simulator declines with a decrease in the output pressure of the ventilator, until the EPAP.
3. As can be seen, the air pressure in the lung simulator always lags behind the output pressure of the ventilator. The main reason is that the respiratory resistance and compliance block the increase in the air pressure in the lung simulator.
4. It should be noticed that, if the inspiration time is set shorter, the respiratory resistance or compliance is big enough, and then the air pressure in the lung simulator may not reach IPAP.

As seen in Figure 4, the following can be obtained.

1. The simulation results have a good consistency with the experimental results, and this verifies the mathematical model above.
2. In the inspiration process, with an increase in the output pressure of the ventilator, the input air flow of lung simulator rises sharply, but the rise velocity reduces continuously. When the output pressure of the ventilator gets to IPAP, the input air flow of lung simulator starts to decline. And finally the lung simulator stops inspiration when the air pressure in lung simulator is the same as the output pressure of the ventilator.
3. In the expiration process, the output air flow of lung simulator increases sharply with a reduction in the output pressure of ventilator, and the rise velocity reduces constantly until the air pressure in lung simulator tends to be EPAP. When the output pressure of the ventilator sinks to EPAP, the output air flow of lung simulator starts to decline. And finally the output air flow tends to be zero when the air pressure in lung simulator tends to be EPAP.
4. The main reasons for the difference between the experimental results and simulation results are the variation of the respiratory compliance and leakage of the ventilation system. In the simulation, the respiratory compliance is considered as a constant, and it is assumed there is no leakage in the ventilation system. However, the compliance of lung simulator varies with the pressure of lung simulator, and the leakage cannot be avoided in the experimental study.

As shown in Figure 5, the following is clear.

The respiratory resistance fluctuates with time regularly; the fluctuation range of respiratory resistance value, during inspiration, is from 0.72 cm H_2_O/L/s to 3.58 cm H_2_O/L/s. During expiration, it is from 0.72 cm H_2_O/L/s to 3.98 cm H_2_O/L/s.

The variation of the respiratory resistance practically corresponds to the variation of the air mass flow of the lung simulator. However, when the air mass flow tends to zero, based on (7), the respiratory resistance remains almost steady.

## 4. Influence on Air Pressure Dynamic Characteristics

As the air pressure in human lung is very critical to mechanical ventilation treatment, and that is determined by the parameters of ventilation system, for the sake of a good treatment effect, it is necessary to study the influence of the parameters on the pressure dynamic of the ventilation system of the lung simulator.

According to the experimental study and simulation above, each parameter can be changed for comparison while all other parameters are kept constant, and the simulation results varying each parameter are illustrated in Figures 6, 7, 8, 9, 10, 11, 12, 13, and 14.

### 4.1. Influence of Ventilator Settings on the Pressure Dynamic

**Figure 6:**
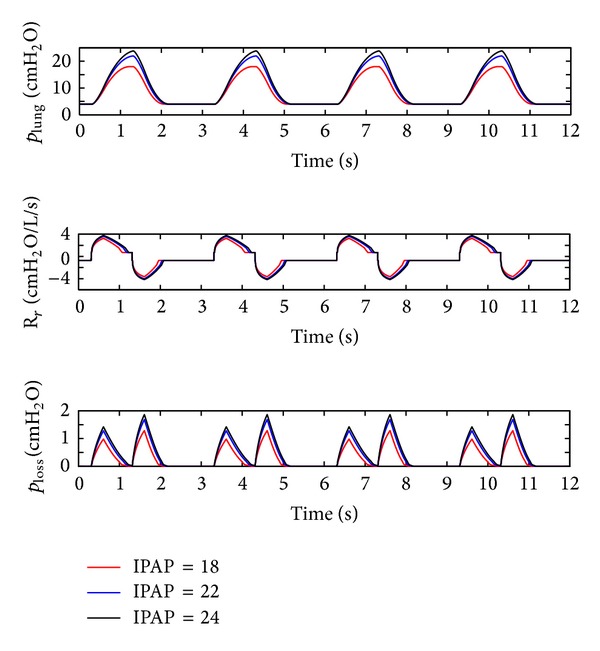
Influence of the IPAP on the air pressure dynamic.

(*1) Influence of the IPAP*. The IPAP of the ventilator is set to 18 cm H_2_O, 22 cm H_2_O, and 24 cm H_2_O, and the simulation results can be seen in Figure 6.

**Figure 7:**
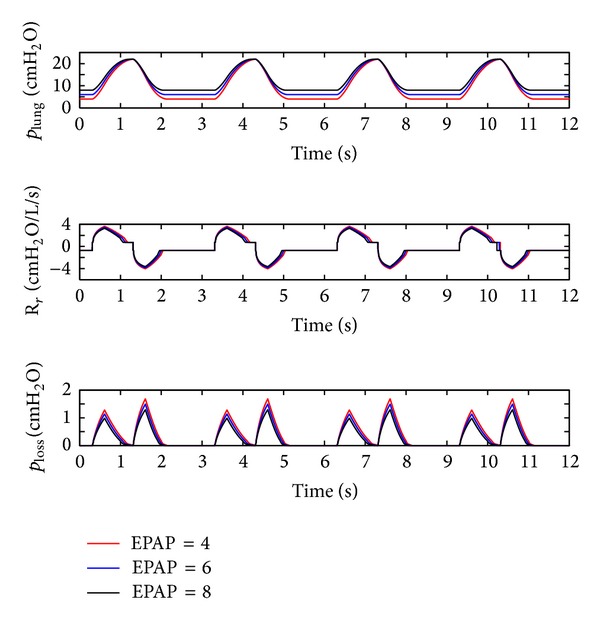
Influence of the EPAP on the air pressure dynamic.

As presented in Figure 6, increasing IPAP may lead to a distinct rise in the pressure (*p*_lung_) of the lung simulator and pressure loss (*p*_loss_) of the ventilation system and a slight growth in maximum respiratory resistance (*R*_*r*_). The fluctuations of the pressure (*p*_lung_) of the lung simulator and the pressure loss (*p*_loss_) of the ventilation system increase with an elevation in IPAP.

(*2) Influence of the EPAP.* The EPAP of the ventilator is set to 4 cm H_2_O, 6 cm H_2_O, and 8 cm H_2_O, and the simulation results are illustrated in Figure 7.

**Figure 8:**
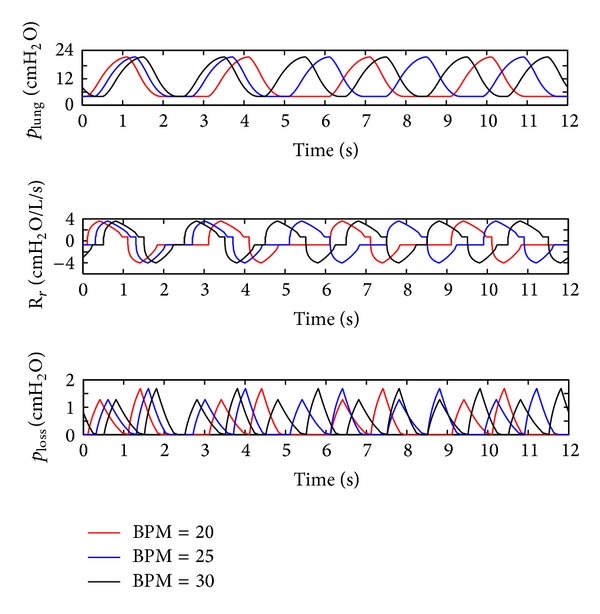
Influence of the BPM on the air pressure dynamic.

As shown in Figure 7, EPAP elevation may result in a distinct rise in the pressure (*p*_lung_) of the lung simulator, a significant drop in the pressure loss (*p*_loss_) of the ventilation system, and a slight reduction in maximum respiratory resistance (*R*_*r*_). When EPAP is set smaller, the fluctuations of the pressure (*p*_lung_) of the lung simulator and the pressure loss (*p*_loss_) of the ventilation system may become bigger.

(*3) Influence of the BPM*. The BPM of the ventilator is set to 20, 25, and 30, while the inspiration time was kept constant, namely, 1 s. The simulation results are shown in Figure 8.

**Figure 9:**
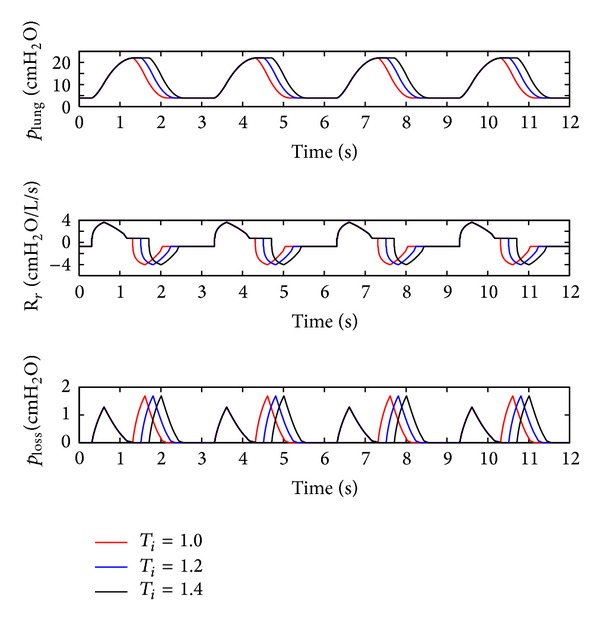
Influence of the *T*_*i*_ on the air pressure dynamic.

As illustrated in Figure 8, BPM just affects the cycle of the ventilation system and its influence on the other dynamics can be neglected.

(*4) Influence of the Inspiration Time *(*T*_*i*_). The inspiration time (*T*_*i*_) of the ventilator is set to 1 s, 1.2 s, and 1.4 s. The simulation results are depicted in Figure 9.

**Figure 10:**
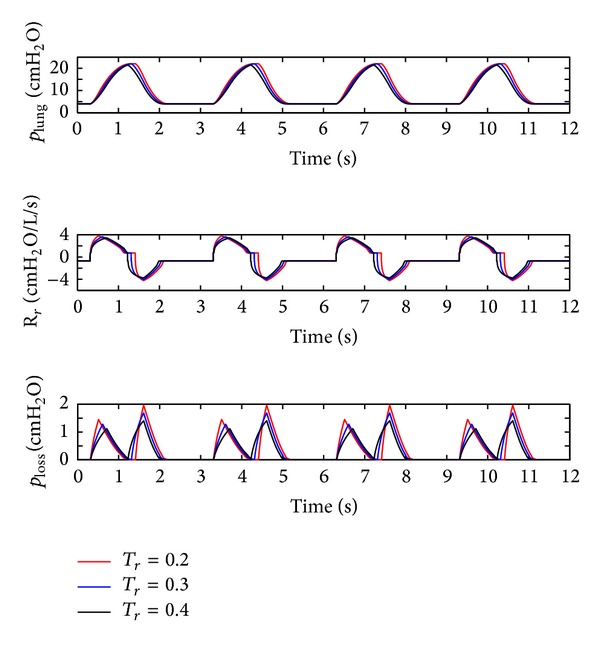
Influence of *T*_*r*_ on the air pressure dynamic.

As shown in Figure 9, *T*_*i*_ only effects the inspiration time of the lung simulator, and its influences on the other dynamics are negligible.

(*5) Influence of the Pressure Rise Time *(*T*_*r*_). The pressure rise time (*T*_*r*_) of the ventilator is set to 0.2 s, 0.3 s, and 0.4 s. The simulation results are shown in Figure 10.

From Figure 10, it is observed that the air pressure (*p*_lung_) in lung simulator and the respiratory resistance (*R*_*r*_) is influenced moderately, but the pressure loss of the system increases distinctively with a rise in the pressure rise time (*T*_*r*_). The main reason is that, at the beginning of the inspiration and expiration, the air flow increases with a decrease in the pressure rise time (*T*_*r*_).

### 4.2. Influence of the Respiratory Compliance (*C*)

**Figure 11:**
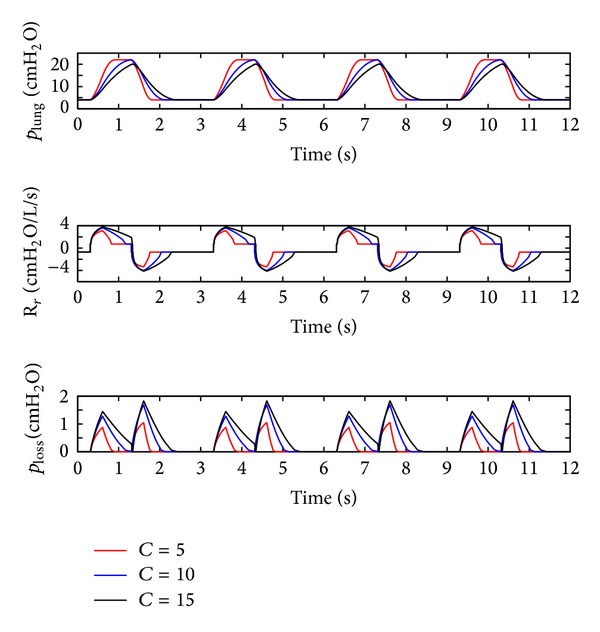
Influence of *C* on the air pressure dynamic.

**Figure 12:**
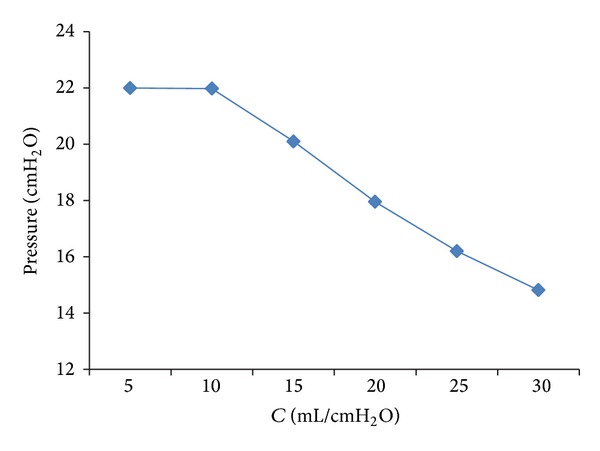
Influence of *C* on the peak pressure of lung simulator.

The respiratory compliance (*C*) of the lung simulator is set to 5 mL/cm H_2_O, 10 mL/cm H_2_O, and 15 mL/cm H_2_O, and the simulation results are illustrated in Figure 11. The relationship between the peak pressure of lung simulator and the respiratory compliance (*C*) is studied, with the results shown in Figure 12. From Figures 11 and 12, the following can be seen.

Firstly, with the decline in the respiratory compliance (*C*), the rise and fall velocity of air pressure in the lung simulator ascend significantly, but the respiratory resistance (*R*_*r*_) and pressure loss of the system descend distinctively.

Furthermore, when the respiratory compliance (*C*) is smaller than 10, the air pressure in the lung simulator can reach IPAP. However, when the *C* is bigger than 10, the peak pressure of the lung simulator is inversely proportional to the respiratory compliance (*C*).

Finally, the amplitude of the respiratory resistance (*R*_*r*_) and the pressure loss of the system go down with a drop in the respiratory compliance (*C*).

### 4.3. Influence of the Diameter (*d*) of the Equivalent Effective Area

**Figure 13:**
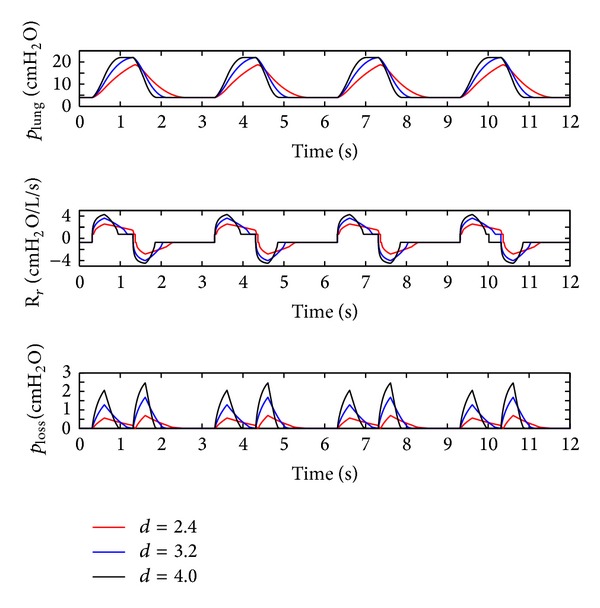
Influence of the diameter (*d*) of the effective area on the air flow dynamic.

**Figure 14:**
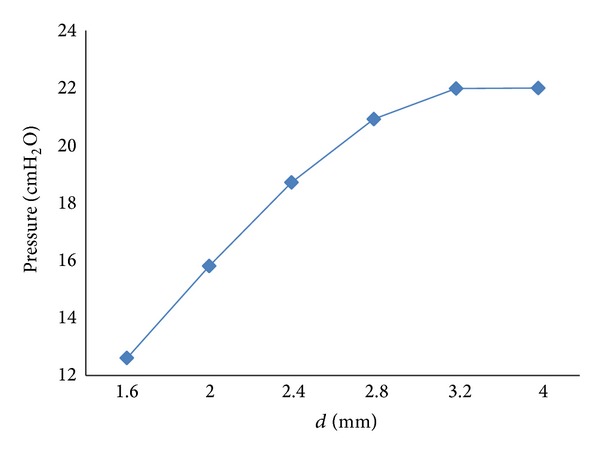
Influence of the diameter (*d*) of the effective area on peak pressure of lung simulator.

As discussed above in Section 2.2.4, the respiratory resistance (*R*_*r*_) is mainly influenced by the diameter (*d*) of the effective area of the equivalent throttle. The diameter (*d*) of the effective area of the throttle is set to 2.4 mm, 3.2 mm, and 4.0 mm, and the simulation results are shown in Figures 13 and 14.

As shown in Figures 13 and 14, the following is obvious.

First of all, with a growth in the diameter (*d*) of the effective area, the rise and fall velocities of air pressure in lung simulator, the respiratory resistance (*R*_*r*_), and pressure loss of the system ascend significantly.

Furthermore, as shown in Figure 14, when the diameter (*d*) of the effective area is larger than 3.2 mm, the air pressure in the lung simulator can reach IPAP. When the diameter (*d*) of the effective area is smaller than 3.2 mm, the peak pressure of the lung simulator increases with a rise in the diameter (*d*) of the effective area.

Lastly, the amplitude of the respiratory resistance (*R*_*r*_) and the pressure loss of the system fall down with a reduction in the diameter (*d*) of the effective area.

## 5. Conclusions

In this paper, the mechanical ventilation system was considered as a pure pneumatic system, and then a new mathematical model of mechanical ventilation system was set up. For the validation of the mathematical model, a prototype mechanical ventilation system of a lung simulator was proposed. Simulation and experimental studies on the air pressure dynamics of the lung simulator were done and the conclusions are summed up as follows.

1. The measured data has a good consistency with the ventilator report, and the experiment is authentic and reliable.
2. The simulation results are consistent with the experimental results, which verify the mathematical model.
3. The air pressure in the lung simulator rises with a growth in the output pressure of the ventilator and declines with a decrease in the output pressure of the ventilator. The air pressure in the lung simulator always lags behind the output pressure of the ventilator.
4. Increasing IPAP may lead to a distinct rise in maximum pressure of the lung simulator. The EPAP elevation may result in a significant rise in minimum pressure of the lung simulator. Influences of BPM, the inspiration time (*T*_*i*_), and the pressure rise time (*T*_*r*_) on the pressure dynamics of the lung simulator are very slight.
5. When the respiratory compliance (*C*) is smaller than 10 mL/cm H_2_O, the air pressure in the lung simulator can reach IPAP. However, when the *C* is bigger than 10 mL/cm H_2_O, the peak pressure of the lung simulator is inversely proportional to the respiratory compliance (*C*).
6. When the diameter (*d*) of the equivalent effective area is larger than 3.2 mm, the air pressure in the lung simulator can get to IPAP. But when the diameter (*d*) of the effective area is smaller than 3.2 mm, the peak pressure of the lung simulator increases with a rise in the diameter (*d*) of the effective area.

The study can be referred to in the respiratory diagnostics, treatment, and design of various medical devices or diagnostic systems. In addition, it may accelerate research on the development of new diagnostic and treatments.

## Figures and Tables

**Table 1:** Values of the main ventilator setting.

	Parameter				
	IPAP (cmH_2_O)	EPAP (cmH_2_O)	BPM	*T*_*i*_ (s)	*T*_*r*_ (s)
Value	22	4	20	1	0.2

## Nomenclature

*A*_*e*_: Effective area of throttle (m^2^)
*b*: Critical pressure ratio = 0.528
*C*: Respiratory compliance (L/cm H_2_O)
*d*: Diameter of effective area (m)
*l*: Length (m)
*m*: Mass of air (kg)
*p*: Pressure (pa)
*q*: Air mass flow (kg/s)
*Q*: Air volume flow (m^3^/s)
*R*: Gas constant = 287 (J/(kg*·*K))
*R*_*r*_: Respiratory resistance (cm H_2_O/L/s)
*T*: Temperature (K)
*t*: Time (s)
*V*: Volume (m^3^)
*λ*: Friction coefficient
*ρ*: Density (kg/m^3^)
*κ*: Specific heat ratio = 1.4.

### Subscripts

1: Equivalent throttle 1
2: Equivalent throttle 2
*d*: Downstream side
*e*: Exhalation valve
lung: Parameter of lung
loss: Pressure loss
*u*: Upstream side
*t*: Tube.
